# DiffSLC: A graph centrality method to detect essential proteins of a protein-protein interaction network

**DOI:** 10.1371/journal.pone.0187091

**Published:** 2017-11-09

**Authors:** Divya Mistry, Roger P. Wise, Julie A. Dickerson

**Affiliations:** 1 Bioinformatics and Computational Biology, Iowa State University, Ames, Iowa, United States of America; 2 Department of Electrical and Computer Engineering, Iowa State University, Ames, Iowa, United States of America; 3 Corn Insects and Crop Genetics Research Unit, USDA-Agricultural Research Service, Ames, Iowa, United States of America; 4 Department of Plant Pathology and Microbiology, Iowa State University, Ames, Iowa, United States of America; Weizmann Institute of Science, ISRAEL

## Abstract

Identification of central genes and proteins in biomolecular networks provides credible candidates for pathway analysis, functional analysis, and essentiality prediction. The DiffSLC centrality measure predicts central and essential genes and proteins using a protein-protein interaction network. Network centrality measures prioritize nodes and edges based on their importance to the network topology. These measures helped identify critical genes and proteins in biomolecular networks. The proposed centrality measure, DiffSLC, combines the number of interactions of a protein and the gene coexpression values of genes from which those proteins were translated, as a weighting factor to bias the identification of essential proteins in a protein interaction network. Potentially essential proteins with low node degree are promoted through eigenvector centrality. Thus, the gene coexpression values are used in conjunction with the eigenvector of the network’s adjacency matrix and edge clustering coefficient to improve essentiality prediction. The outcome of this prediction is shown using three variations: (1) inclusion or exclusion of gene co-expression data, (2) impact of different coexpression measures, and (3) impact of different gene expression data sets. For a total of seven networks, DiffSLC is compared to other centrality measures using *Saccharomyces cerevisiae* protein interaction networks and gene expression data. Comparisons are also performed for the top ranked proteins against the known essential genes from the *Saccharomyces* Gene Deletion Project, which show that DiffSLC detects more essential proteins and has a higher area under the ROC curve than other compared methods. This makes DiffSLC a stronger alternative to other centrality methods for detecting essential genes using a protein-protein interaction network that obeys centrality-lethality principle. DiffSLC is implemented using the igraph package in R, and networkx package in Python. The python package can be obtained from git.io/diffslcpy. The R implementation and code to reproduce the analysis is available via git.io/diffslc.

## Introduction

**Fig 1:**
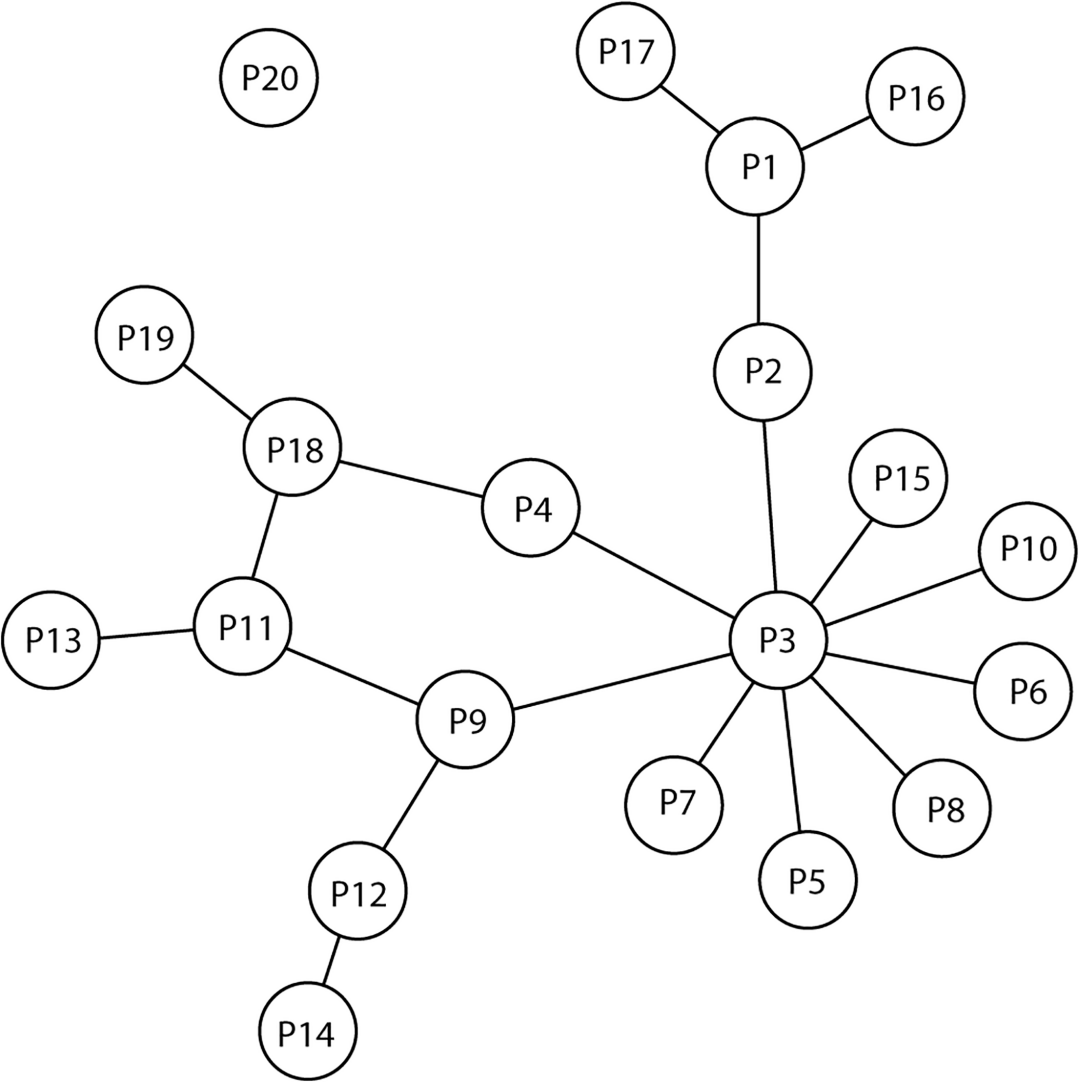
A toy example of a PPI network. A hypothetical PPI network with twenty proteins and their interaction partners. The protein P20 does not have an interaction partner.

With the rise of reliable high-throughput data, computational methods that predict essential genes using protein interaction and gene expression data have shown some promise [1–6]. Protein interaction data, such as those derived from yeast two-hybrid (Y2H) [7, 8], affinity chromatography [9], co-immunoprecipitation [10, 11] etc. can be used to create a protein-protein interaction (PPI) network. In a PPI network, nodes represent proteins, and edges connecting those proteins indicate interaction partners. Fig 1 shows a toy PPI network with twenty proteins P1 to P20. Among these proteins, P1 to P19 have interaction partners, whereas P20 does not interact with any other proteins. Jeong et.al. [1] established the centrality-lethality hypothesis in yeast protein-protein interaction networks, which state that gene knockouts of genes representing hub proteins (i.e. a protein interacting with many other proteins) in a protein-protein interaction network are more likely to be lethal for the organism. Raman et.al. [12] further verified this finding in over fifteen organisms. For detection of essential genes and proteins, much research effort has been focused on finding a single measure that can optimally rank essential proteins based on network topology. Where the centrality-lethality hypothesis is partially helpful, a stronger essentiality predictive centrality metric has been a challenge.

Of the many ways available to select nodes of importance in a biological network, node centrality analysis provides a fast and reproducible way to rank the nodes based on their location in a network. A node ranking method designed to look for essential proteins should give higher rank to essential proteins and lower rank to non-essential ones. These network centrality measures often use path length-based or random walk-based metrics to calculate a node’s relative importance within a network. Closeness, betweenness, node clustering coefficient, and average path length centrality are examples of such measures. More recent approaches have utilized eigenvalues [13] and spectra of the adjacency matrix of a graph [14] to rank nodes. Kim et.al. [15] concluded that shortest-path betweenness was a useful measure to detect genes that are more likely to acquire essentiality in another organism through orthology; however, once an essential function was acquired, the genes were again likely to follow the centrality-lethality principle in the new organism. He and Zhang [16] also showed that betweenness and closeness by themselves are not predictive of protein essentiality in a protein protein interaction network. Subgraph centrality provides good estimates for ranking based on closely connected network neighborhoods, but its ability to predict protein essentiality in a yeast PPI network has not exceeded the performance of degree centrality.

There are several methods that utilize multiomics data to aid essentiality detection algorithms. When additional forms of high-quality experimental data such as protein interaction affinities, canonical gene regulatory networks, metabolic networks, protein co-localizations, RNAi screens, etc. are available for an organims, they can be utilized as essentiality predicting feature vectors for a machine learning algorithm [17] or as participants in a consensus building ensemble methods [18]. More recently, LBCC [19] and Plaimas’ support vector machine (svm) based method [20] have shown significant improvement in essentiality prediction given the various types of high-quality experimental data mentioned above. LBCC used protein complex interaction data along with protein interaction propensities to implement a hypothesis that essential proteins tend to maintain their interaction complexes through the course of evolutionary time scales. ION [21] utilized the same hypothesis to propose an improvement based on known orthologs of an organisms. Plaimas et. al.’s method utilized codon recurrences, phyletic retention, silent site codon composition, and over twenty other metabolic network derived features. For an organism or a specific mutant where high-quality multiomics data sets are available, Zhang et. al.’s expansive review [17] for essential prediction methods may serve as a useful reference.

Until an affordable and reliable high-throughput experiment is available for essentiality detection, a computational approach that can utilize currently available protein interaction and gene expression data can narrow down candidates to test for essentiality.

The proposed DiffSLC method combines multiple centralities. An investigation in combining multiple centralities to benefit from strengths of each is underexplored. DiffSLC is aimed at finding essential proteins in a PPI network using graph topological features as well as experimental data. Based on the results showing success of centrality-lethality principle for PPI networks [1, 12, 15], DiffSLC uses gene expression data to bias degree centrality towards interacting proteins that have similar expression profiles from a transcript-based context. DiffSLC exploits the advantages of eigenvector centrality and edge clustering coefficients. Eigenvector centrality provides higher ranks to low-degree nodes that are connected to high degree nodes, while the edge clustering coefficient ranks graph edges based on their involvement in closely connected small subnetworks [22].

DiffSLC and related analyses are tested with a PPI network and gene expression data from yeast, because high-throughput and reliable experimental data are readily available, and a reference essential genes list is available from the Database of Essential Genes (DEG) [23]. The DEG curates results from single-gene knockout experiments reported in *Saccharomyces* Gene Deletion Project [24]. DiffSLC is aimed at finding essential proteins in a protein-protein interaction network using graph topological features as well as experimental data.

## Materials and methods

The DiffSLC measure uses eigenvector centrality to capture low-degree nodes, and biases node degree computation towards locally central interaction edges and highly coexpressed probesets corresponding to those interactions. The analysis is done on two different sets of yeast microarray experiments, which are combined with a yeast PPI network. Experimental data collection, processing, and analysis are performed as described below.

### Experimental data

Graph centrality analysis was performed on an undirected network, where centrality calculations were biased towards proteins that interact and have very similar expression patterns. To test the effect of different gene expression data sets and their contribution in bias levels, two *Saccharomyces cerevisiae* (yeast) experiments were used. The data sets described below were used to create the network. This rich set of experimental data helped investigate the impact of combining modifications of eigenvector and degree centrality biased by gene coexpression levels, and impact of different gene expression datasets on centrality ranking presented here.

#### Protein-protein interaction data

Protein-protein interaction data for yeast was obtained from the Database of Interacting Proteins (DIP) [25, 26] Release Scere20150101. The interactions were downloaded in PSI-MI MITAB v2.5 format [27]. The tab delimited file was processed in R [28] using the built-in delimited data table processing function. The data provided by DIP included interactions of yeast proteins with other organisms as well. To create a yeast-specific protein interaction network, the non-yeast interactions were removed. After this removal and removing redundant interactions, the resulting interaction data included 5022 proteins and 22860 interactions.

Additional processing was done to remove interactions where between one and three proteins interacted among themselves, but not with any other proteins. Such interactions create a one or two edge subnetwork that is disconnected from rest of the PPI network. Without additional experimental data linking them to rest of the network, such interactions would not add any useful information for our proposed method, and therefore they were also removed from the interaction data. This resulted in 4,958 proteins having 22,308 interactions.

#### Gene expression data

A well-studied yeast gene expression dataset by Tu et.al. [29] (GEO accession GSE3431) highlights the cyclic nature of yeast gene expression in a wild-type yeast strain. Tu et.al. performed a whole genome RNA microarray study to understand the yeast metabolic cycle over 36 time points between approximately 66 to 80 hours after exposure to a nutrient-limited condition. They showed that over half of yeast genome was being expressed at regular intervals during yeast metabolic cycles. The experiment was conducted on an Affymetrix GeneChip Yeast Genome S98 Array. This array had 9,335 probesets which mapped to 6,777 genes. A total of 36 GeneChips were used to profile the gene expression of yeast from continuous culture under nutrient-limited environment. Samples were taken approximate 25 minutes apart.

To understand the impact of using different gene expression datasets on the centrality calculation, a different yeast gene expression dataset was chosen. This experimental data was generated by Guan et al. [30] (GEO accession GSE3076). In their time-series experiment, Guan et al. studied the role of nonsense-mediated mRNA decay (NMD) in a budding yeast global gene expression profile. The NMD pathway in eukaryotes targets transcripts with premature stop codons to eliminate translation of potentially harmful proteins [31]. Guan et al.’s experiments observed that a significant subset of all mRNA produced in a cell were targets of NMD processes. 45% ± 5% of those were direct targets, and 30% of the protein-coding targets affected either chromosome structure and behavior, or cell surface dynamics. The experiment was a time series experiment performed at 16 time-points within the first hour of transcription inhibition signal. The expression profiles were estimated on Affymetrix GeneChip Yeast Genome S98 array.

#### Gene expression data processing

For each of the microarray experiments, corresponding CEL files were processed using the affy [32] package in R/Bioconductor [33, 34]. Additional pre-processing was done to obtain RMA [35] expression values. Relevant data and R programs are available at http://git.io/diffslc.

#### List of essential proteins

The *Saccharomyces* Genome Deletion Project (SGDP) [24, 36] used a PCR-based gene deletion strategy to delete all known ORFs from their start codon to stop-codon. At the completion of the project, a list of 1,156 essential ORFs was produced, which is available at http://www-sequence.stanford.edu/group/yeast_deletion_project/Essential_ORFs.txt. The Database of Essential Genes (DEG) [37] has curated that list over time, and has provided 1,110 currently accepted known essential yeast genes along with relevant features and gene name synonyms in their latest release available for download (DEG v10) [23]. Although a subset of the remaining ORFs and corresponding genes have undetermined essentiality, all the genes that weren’t specifically in the list of 1,110 essential genes from DEG, are assumed non-essential to ensure a conservative estimate of success.

#### DIP interactor to gene name mapping

The data from DIP was provided with the interactor IDs, and their corresponding UniProtKB and Ensembl IDs, if available, at the time of DIP data release. Of the 4958 interacting proteins, 71 proteins did not have a corresponding verified ID in UniProtKB. For the unmatched DIP interactor IDs, a corresponding Ensembl ID was matched and saved. The Affymetrix supplied annotations (NetAffx Annotation Release 35) for the GeneChip Yeast Genome S98 array and GeneChip Yeast Genome 2.0 array provide affy probeset ID to UniProtKB/SwissProt and Ensembl ID mapping. By combining the DIP ID to UniProtKB/Ensembl ID mapping, with Affymetrix annotations, the DIP interactor ID to probeset ID mappings were obtained. There were cases where multiple probesets mapped to a single DIP interactor ID. In such a case, the probeset with lowest expression was mapped to the DIP interactor ID. This was a conservative estimate, but a useful one because it could assumed that any of the candidate transcripts would have undergone at least that lowest amount of transcription for the production of a given protein. The original and filtered data are available along with the source code at http://git.io/diffslc.

### Gene co-expression and graph centrality measures

Much of the publicly available high-throughput protein interaction data utilize experimental techniques prone to high false positive rates [38]. Use of gene expression data in conjunction with protein interactions has yielded improvements in essentiality prediction [39, 40]. The method proposed by Li et al. [39], and later verified by Tang et al. [40], improved essentiality predictability by using edge clustering coefficients combined with Pearson correlation between coexpressed probesets as edge weight.

#### Protein-protein interaction network

Using the filtered DIP data described in previous section, an undirected network was created using the DIP interactors as nodes and their interactions as edges. This was an unweighted protein interaction network. Based on the centrality-lethality hypothesis, node degree was expected to be predictive of protein essentiality in this network. Wang et.al. [41] showed that using the edge clustering coefficient values (ECC) as edge weights in a protein interaction network substantially improves essentiality prediction. The ECC of the protein interaction network will be used as a contributor to the gene coexpression bias for centrality calculation.

The following two sections define all the gene co-expression measures used for biasing centrality calculations, the variety of centrality measures compared in this study, and the edge clustering coefficient defined by [22]. The proposed DiffSLC is also defined at the end of the section.

#### Gene co-expression measures

To use the gene expression data from Tu et.al. [29] and Guan et.al. [30] in node centrality calculation, various gene coexpression measures were compared to understand the effect of choosing different coexpression metrics. For genes *X* and *Y*, the following methods can be used to calculate each of the co-expression measures.

$$\begin{array}{c} X=\left(x_{1},\ldots ,x_{m}\right),Y=\left(y_{1},\ldots ,y_{m}\right) \end{array}$$

where *X*, *Y* are arbitrary genes, and *x*_*i*_, *y*_*i*_ are *i*th observed expression values.

- **Pearson correlation coefficient:** This measure assumes normal distribution of *X* and *Y*, and estimates a monotonic relationship between the variables. R’s built-in implementation of Pearson correlation is used for this computation.
  $$\begin{array}{c} pCor\left(X,Y\right)=\frac{\sum_{i=1}^{m}\left(x_{i}-\bar{x}\right)\left(y_{i}-\bar{y}\right)}{\sqrt{\sum_{x_{i}}^{m}{\left(x_{i}-\bar{x}\right)}^{2}}\sqrt{\sum_{y_{i}}^{m}{\left(y_{i}-\bar{y}\right)}^{2}}} \end{array}$$
  where $\bar{x}$, $\bar{y}$ are sample means for *X*, *Y* respectively.
- **Spearman’s rank correlation coefficient:** This non-parametric estimator does not make assumptions about distributions of *X* and *Y*, and estimates monotonic association between the variables. R’s built-in implementation of Spearman’s rank correlation is used for this computation.
  $$\begin{array}{c} sCor\left(X,Y\right)=1-\frac{6\;\sum_{i=1}^{m}d_{i}^{2}}{m(m^{2}-1)} \end{array}$$
  where, *d*_*i*_ is the difference between ranks of *x*_*i*_ and *y*_*i*_.
- **Distance correlation:** This measure estimates a non-monotonic relationship between *X* and *Y* random variables. A desirable feature of this measure is that a zero distance correlation implies variable independence [42, 43]. The distance correlation implemented in the energy [44] package for R is used for this computation. The distance correlation (*dCor*) between *X* and *Y* is defined in terms of distance covariance (*dCov*) and distance variance (*dVar*) of both variables. In the following formula, *f*_*X*_ and *f*_*Y*_ are the characteristic functions of *X* and *Y* respectively, while *f*_*X*,*Y*_ is the joint characteristic function.
  $$\begin{array}{ccc} dCor(X,Y) & = & \frac{dCov(X,Y)}{\sqrt{dVar(X)}\sqrt{dVar(Y)}}\\ & = & \frac{∥f_{X,Y}\left(t,s\right)-f_{X}\left(t\right)f_{Y}\left(s\right)∥}{\sqrt{∥f_{X,X}\left(t,s\right)-f_{X}\left(t\right)f_{X}\left(s\right)∥}\sqrt{∥f_{X,Y}\left(t,s\right)-f_{X}\left(t\right)f_{X}\left(s\right)∥}} \end{array}$$

#### Centrality measures

Graph centrality measures are used to provide relative importance ranking to nodes and edges of a network. For the following centrality definitions, a network is defined as follows. Let *G* be an undirected weighted graph, where *V* is a set of *N* vertices (or nodes), and *E* is a set of edges (or links).

$$\begin{array}{ccc} G & = & (V,E),\\ \left|V\right| & = & N\\ E & = & \left\{(u,v,w)|u\in V,v\in V,u\neq v,|(u,v)|=w\right\} \end{array}$$

- **Edge clustering coefficient:** The edge clustering coefficient is defined as the number of triangles, which include the given edge divided by the number of triangles the edge may participate in based on the node degree of its incident nodes. For the graph *G*, the edge clustering coefficient (ECC) of an arbitrary edge *e* connecting nodes *u* and *v* can be calculated using the following method in [22].
  $$\begin{array}{c} ECC_{u,v}^{\left(3\right)}=\frac{z_{u,v}^{\left(3\right)}+1}{min[\left(k_{u}-1\right),\left(k_{v}-1\right)]} \end{array}$$
  Where $z_{u,v}^{\left(3\right)}$ is the number of triangles including the edge *e*, and *k*_*u*_, *k*_*v*_ are number of triangles *u*, *v* participate in.
- **Degree centrality:** The degree centrality of a node is the number of its adjacent edges in a graph. For a directed graph, this number can be separated into a node’s “in degree” and “out degree” referring to the number of edges coming into the node or going away from the node, respectively. For undirected graphs, a node’s degree is the number of other nodes it is linked to. For the graph *G*, degree centrality (DC) of an arbitrary node *u* can be calculated using the following formula.
  $$\begin{array}{c} DC(u)=|(u,\cdot ,\cdot )\in E| \end{array}$$
- **Shortest path closeness centrality:** The closeness is defined as an inverse of farness, where a node’s farness is defined as sum of all the shortest path lengths between it and other nodes. This shortest path-based measure was introduced by Sabidussi [45]. A random-walk based modification of Sabidussi’s closeness centrality measure was suggested by Noh and Rieger [46]. Both variations of closeness centrality have been used to understand topology of biological networks. In essence, the higher the closeness of a node, the *quicker* it is to reach other nodes from that node. For the graph *G* defined earlier, the shortest path closeness centrality (CC) of an arbitrary node *u* can be calculated using the following formula.
  $$\begin{array}{c} CC\left(u\right)=\sum_{u\neq v}\;\frac{1}{d(u,v)} \end{array}$$
  where *d*(*u*, *v*) is the shortest-path distance between nodes *u* and *v*.
- **Shortest path betweenness centrality:** A node that lies along the shortest paths between many pairs of nodes is considered more important than a node with fewer shortest paths passing through it. This notion is referred to as the betweenness of a node within a graph. The shortest path-based betweenness centrality measure was introduced by Freeman [47], and it was later modified as a random-walk based measure by Newman [48]. For the graph *G* defined earlier, the shortest path between centrality (BC) of an arbitrary node *u* can be calculated using the following formula.
  $$\begin{array}{c} BC\left(u\right)=\sum_{\underset{i,u,j\in V}{i\neq u\neq j}}\;\frac{\sigma_{ij}\left(u\right)}{\sigma_{ij}} \end{array}$$
  where *σ*_*ij*_ is the number of shortest paths between nodes *i* and *j*, and *σ*_*ij*_(*u*) is the number of those paths that pass through node *u*.
- **Eigenvector centrality:** The values of the eigenvector corresponding to the greatest eigenvalue of the graph’s adjacency matrix is used as nodes’ centrality score [49], called the eigenvector centrality. The popular PageRank™ [50] algorithm used by Google™Search is a modification of eigenvector centrality, where the eigenvalue of interest is calculated using a power iteration method. In essence, eigenvector centrality score ranks nodes based on how many other high ranking nodes a given node connects to. For the graph *G* defined earlier with the adjacency matrix *A*, the eigenvector centrality (EC)—assumed to be a positive number —can be calculated using the following method described by Newman in [13].
  Let *x*_*u*_ be the EC of vertex *u*.
  $EC(u)=x_{u}=\frac{1}{\lambda }\sum_{j=1}^{n}\;A_{uj}x_{j}$, where λ is a constant.
  Let **x** = (*x*_1_, *x*_2_, …) be the vector of EC’s.
  This gives us λ**x** = **A** ⋅ **x**.
  Thus, **x** is an eigenvector corresponding to the largest eigenvalue λ of **A**.
- **Subgraph centrality:** The subgraph centrality measure was introduced by Estrada and Rodriguez-Velazquez [14]. It quantifies the influence of a node in a subgraph of the given graph using the spectra of the given graph’s adjacency matrix. This measure gives more weight to smaller subgraphs than larger ones, thus making it a good measure for understanding network motifs. For the graph *G* defined earlier, the subgraph centrality (SC) of an arbitrary node *u* can be calculated using the following steps described in [14].
  Let λ_1_, λ_2_, …, λ_*n*_ be the eigenvalues of **A**, the adjacency matrix.
  Let $\nu_{j}^{i}$ be the *i*th component of the *j*th eigenvector associated with λ_*j*_ eigenvalue.
  Then, $SC\left(u\right)=\sum_{j=1}^{N}{\left(\nu_{j}^{u}\right)}^{2}\exp^{\lambda j}$
- **DiffSLC:** DiffSLC is defined as a weighted combination of the eigenvector centrality and the coexpression-biased degree centrality. While degree centrality is able to capture many of the essential proteins in the top 20% of degree sorted nodes, it also misses several known essential proteins with fewer interactions within the DIP interaction dataset. Many of these low-degree nodes are connected to other higher degree nodes. Eigenvector centrality (EC) ranks such nodes higher; hence DiffSLC captures additional essential proteins by giving partial weight to nodes ranked highly by EC.
  Furthermore, the co-expression bias for each pair of interacting proteins is weighted by the coexpression amount and by the edge-clustering coefficient. The coexpression bias detects interacting proteins that are also highly co-expressed in a given gene expression condition. The edge clustering coefficient (ECC) bias promotes protein interactions that may affect other interactions of its interacting proteins, or be affected by other interactions of its interacting proteins. These contributions are captured here via the *β* and *ω* parameters, which vary the levels of contributions from each set of experimental data and centralities. S2 Table shows the results of varying both parameters.
  For the graph *G* defined earlier, the DiffSLC of an arbitrary node *u* can be calculated as follows, where the *BDC*(*u*) is the biased degree centrality of a node *u*.
  $$\begin{array}{c} BDC\left(u\right)=\sum_{i=1}^{m}\;[\left(\beta *dCor\left(u_{i}\right)\right)+\left(\left(1-\beta \right)*ECC\left(u_{i}\right)\right)] \end{array}$$
  where, *u* has *m* incident edges, and *β* ∈ [0, 1].
  $$\begin{array}{c} DiffSLC(u)=(\omega *EC(u))+((1-\omega )*BDC(u)) \end{array}$$
  where, *EC* is the eigenvector centrality, and *ω* ∈ [0, 1].

In this case, *BDC* is a weighted using the distance correlation. For both of the datasets used in the analysis, gene expression profiles are better estimated using distance correlation metric compared to either of the linear correlation metrics. For a case where a monotonic relationship can better estimate the gene coexpression, Spearman’s Rank correlation (*sCor*) may be a more appropriate replacement for *dCor*. Additional details are provided in the Discussion section.

#### Performance estimates

For the yeast dataset, where both positive and negative samples of protein essentiality exist, a node centrality method designed to prioritize essential proteins can be treated as a binary classifier. A perfect prioritization method would rank the essential yeast proteins at the top of its ranking, and rank the non-essential yeast proteins lower in the list. The following three metrics can be used to estimate the performance of the ranking method: Receiver Operating Characteristic (ROC) curve, area under the ROC curve (AUC of ROC), and Precision-Recall (P-R) curve.

- **Receiver Operating Characteristic (ROC) curve:** The ROC curve shows relationship between True Positive Rate (TPR) and False Positive Rate (FPR) for a binary classifier. These rates can be obtained from a 2x2 confusion matrix as shown in Table 1.

**Table 1:** 2x2 confusion matrix.

	Known positive	Known negative
**Predicted positive**	True Positive (TP)	False Positive (FP)
**Predicted negative**	False Negative (FN)	True Negative (FN)

**True Positive** is the number of correctly identified positive samples. **True Negative** is the number of correctly identified negative samples. **False Positive** is the number of negative samples identified as positive, and **False Negative** is the number of positive samples identified as negative by the given method.

Using the confusion matrix, TPR and FPR are calculated as below.

$$\begin{array}{ccc} TPR & = & \frac{TP}{TP+FN}\\ FPR & = & \frac{FP}{FP+TN} \end{array}$$

The canonical representation of the ROC curve is plotted with 1 − *FPR* on the x-axis and *TPR* on the y-axis.

For the ROC curve, the area under the curve (**AUC of ROC**) is estimated using a trapezoid area computation algorithm described in [51]. For a perfect binary classifier, the AUC of ROC curve would be 1. If a sample instance is randomly chosen, the area under the ROC curve represents the probability that the selected random sample is ranked higher if it is a positive instance, and ranked lower if it is a negative instance.

- **Precision-Recall (P-R) curve:** A P-R curve shows a relationship between precision (also known as positive predicted value), and recall (also known as true positive rate or sensitivity). These values can be computed from the 2x2 confusion matrix shown in Table 1.
  For clarity, precision and recall variables defined in the **ROC** section above, are reused here.
  $$\begin{array}{ccc} Precision & = & \frac{TP}{TP+FP}\\ Recall & = & \frac{TP}{TP+FN} \end{array}$$
  The canonical representation of P-R curve is plotted with *Recall* on x-axis and *Precision* on y-axis.

## Results

The DiffSLC was evaluated via the ROC curve, the AUC of the ROC, and the P-R curve. The ROC curve and the AUC of the ROC curve help determine effectiveness of DiffSLC as a discrimination method for essential versus non-essential proteins of each network. The P-R curve estimates the extent to which DiffSLC is able to provide useful results at high false negative, or in other words, detecting essential proteins within top few percent of DiffSLC ranked proteins.

Once the cleaned and curated data for our yeast PPI network were ready, the association of node degree to protein essentiality as suggested by centrality-lethality hypothesis for a PPI network was verified. Additional graph centrality measures were applied to the network to quantify their predictability of protein essentiality for a yeast PPI network. Without further modifications, degree centrality (DC) provided the best predictability (AUC of ROC = 0.64) for protein essentiality as compared against betweenness (BC), closeness (CC), eigenvector (EC), and subgraph (SC) centralities. Betweenness had been hypothesized as a measure predictive of detecting genes acquiring essentiality evolved through orthology [15], and for those predicted essential genes, the centrality-lethality hypothesis was shown to be an identifying feature of essential proteins for a PPI network in yeast and mice. Given a better predictability of DC compared to other centralities, gene co-expression measures were used to bias the degree calculation for each node. EC based ranking had ranked low-degree essential protein nodes highly. This made EC an ideal candidate to combine in DiffSLC. A co-expression biased DC combined with EC, thus, was determined to be able to detect critical nodes of a network that either DC or EC would have missed on their own.

The performance of DiffSLC was compared against the commonly used centralities for PPI network node prioritization. The number of essential proteins detected by all centrality methods in the top 1% to 25% were also reported to highlight the advantage of each method. Comparisons were performed between PPI network without biasing factors (i.e. gene co-expression values and edge clustering coefficients) and networks with the biasing factors. Contribution weights (i.e. values of *β* and *ω*) were chosen based on S2 Table to compare against the optimal cases.

There were 7 different networks generated for analysis and comparison of the proposed method. Table 2 lists these networks and their properties relevant to DiffSLC results. A ✓ indicates the property included in a network.

**Table 2:** Networks being tested for DiffSLC.

	Network ID	pCor	sCor	dCor	ECC	EC
PPI	N0	–	–	–	–	–
PPI + Tu2005	NT1	✓	–	–	✓	✓
PPI + Tu2005	NT2	–	✓	–	✓	✓
PPI + Tu2005	NT3	–	–	✓	✓	✓
PPI + Guan2006	NF1	✓	–	–	✓	✓
PPI + Guan2006	NF2	–	✓	–	✓	✓
PPI + Guan2006	NF3	–	–	✓	✓	✓

**PPI**—a network created from only DIP data. **PPI + Tu2005**—using gene expression data from [29] to bias the centrality calculation for PPI. **PPI + Guan2006**—using gene expression data from [30] to bias the centrality calculation for PPI. The column titles of the table indicate which biasing factors were used to weight edges for DiffSLC computation.

**Fig 2:**
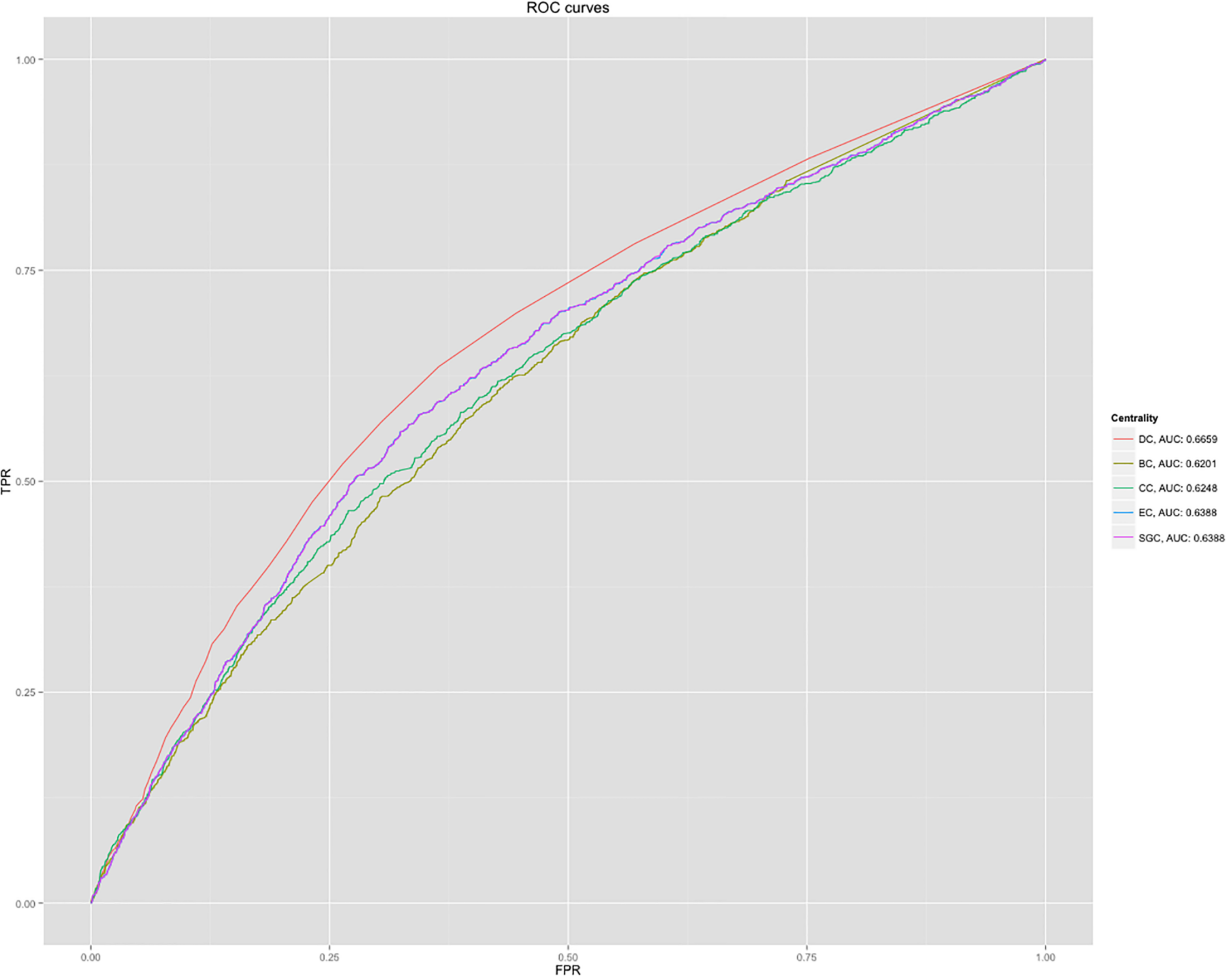
N0_ROC: ROC curves for using various centralities as essentiality predictors. ROC curves of DC, CC, BC, EC, and SC are plotted along with their corresponding AUC.

The centrality-lethality hypothesis suggests that a protein with many interaction partners is more likely to be an essential protein than a protein with fewer interactions; assuming that removal of such central protein would disrupt an organism’s growth. Therefore, in a protein interaction network, nodes with higher degree centrality are more likely to correspond to essential genes. To assess this suggestion, the node degree from network N0 was used as a predictor of protein essentiality. Fig 2 shows the ROC curve of node degree’s ability to predict gene essentiality. Also compared were CC, BC, EC, and SC for the same network.

Fig 2 results show that the best results are generated using DC. The centrality-lethality principle has been observed in yeast multiple times, and this result shows that the data being utilized for DiffSLC analysis also conforms to that expectation.

Over 70 essential genes were low degree nodes in the N0 centrality ranking results, which were within the top-25% of eigenvector centrality (EC) ranked nodes. In other words, these nodes were missed by the top-25% DC ranked nodes; however, EC was able to rank them higher. Other centralities also detected the low degree nodes at varying amounts.

### DiffSLC, and the effect of different co-expression measures

**Fig 3:**
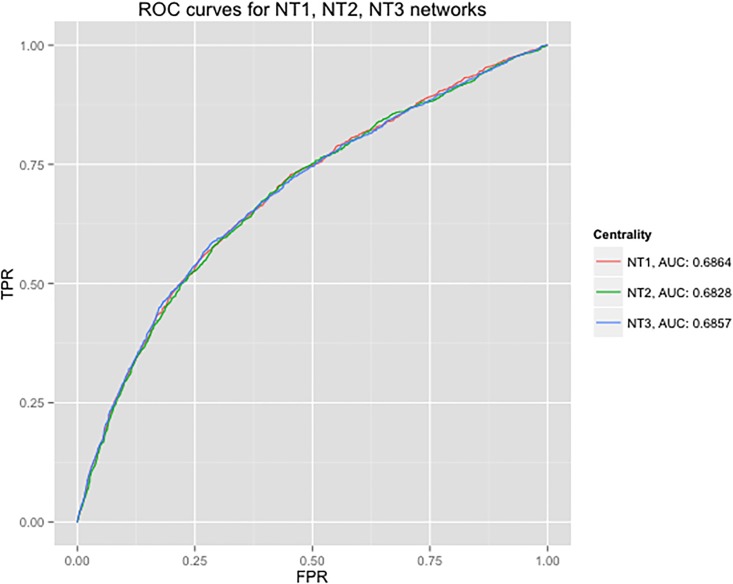
NT1,2,3_ROC: ROC curves for using various gene coexpression measures as biasing factors in DiffSLC. ROC curves of NT1, NT2, and NT3 networks are plotted along with their corresponding *β*, *ω*, and AUC. The differences in AUC is negligible.

Degree centrality was chosen as the base implementation to improve on the results seen in the analysis of network N0. Li et.al. [39] have shown usefulness of the Pearson correlation coefficient in estimating pairwise gene coexpression for the Tu et.al. experiment [29]. Three networks–NT1, NT2, and NT3–were generated to understand the effect of three common gene coexpression measures as described in Methods. The NT1 network was generated by assigning the Pearson correlation of the gene expression values from the Tu et.al. experiment as the edge weights for the N0 network. The other two networks, NT2 and NT3, were generated similarly using the Spearman’s rank correlation and the distance correlation of gene expression, respectively. For networks NT1, NT2, and NT3 corresponding to the Tu et.al. dataset, Fig 3 shows the ROC comparisons of calculating DiffSLC with biased degree centrality (BDC) based on ECC and corresponding co-expression of adjacent edges to a given node. The ROC was calculated on DiffSLC function defined earlier. For these networks, DiffSLC was computed using *β* = 0.8, *ω* = 0.1. DiffSLC was tested by varying *β*, *ω* in the [0.05, 1.00] range. The specific values were chosen based on the best AUC of the ROC reported for each. Different co-expression measures required different *β* and *ω* values in some cases. The best results from each of the co-expression measures are provided in Fig 3.

Fig 3 shows that the impact of choosing different coexpression measures is negligible for the Tu et.al. data set. In their analysis, Tu et.al. showed that the expression of genes follows periodic metabolic cycles, with around 9 cycles for their experimental result. Because a distance correlation measure is designed to estimate non-monotonic relationship between two random variables, it is better suited for this method. Keeping that in mind, the NT3 network will be used to compare against the the chosen network from the NF1,2,3 networks.

**Fig 4:**
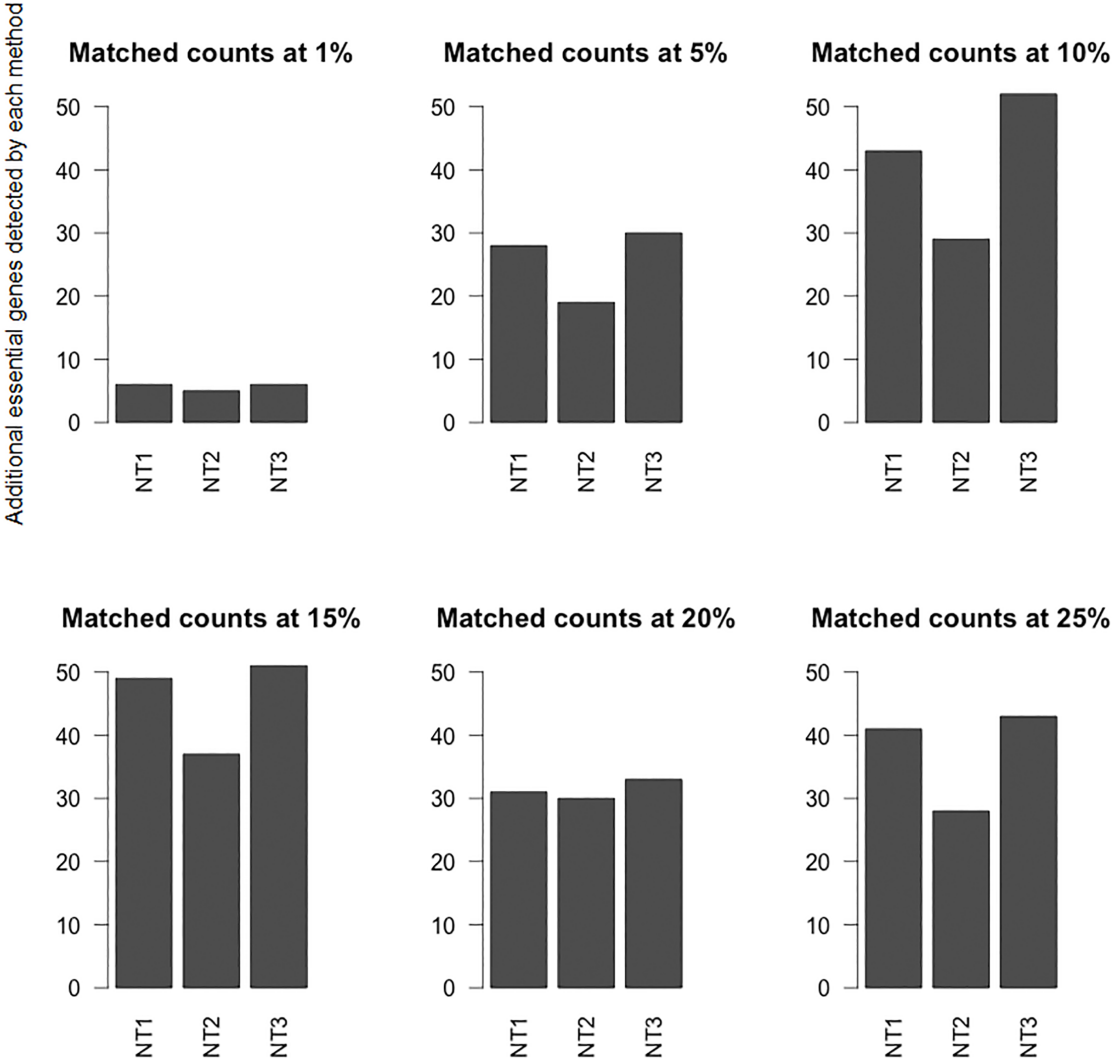
NT1,2,3_Top ranked proteins. Counts of unique essential genes ranked by various DiffSLC variants in the top 1%, 5%, 10%, 15%, 20%, and 25% are shown in the plot. These counts are only for a set of genes that were detected by one of the networks but not the others.

The results of *Saccharomyces* Genome Deletion Project [24] suggested that less than 20% of yeast genes were essential genes. As such less than top 25% of the ranked proteins in the yeast networks are of interest. These top ranked nodes of the network should provide a targeted list of candidates to be considered for essentiality verification. Results from Fig 3 suggest that the number of detected essential proteins may not be very different; however, for experimental verification, even a difference of 20–30 gene knockout candidates can make a big difference. Fig 4 shows unique matched counts at various percentage level. These counts are the number of essential proteins detected by each ranking method in the top 1–25% that the other methods did not detect.

Fig 4 shows that at each of the percent cutoff levels, the NT3 network marginally outperforms the NT1 network in detection of more essential genes. This reaffirms the choice of NT3 as a reasonable choice to compare against the chosen one among the NF1,2,3 networks.

### DiffSLC, and the effect of different gene expression experiment

**Fig 5:**
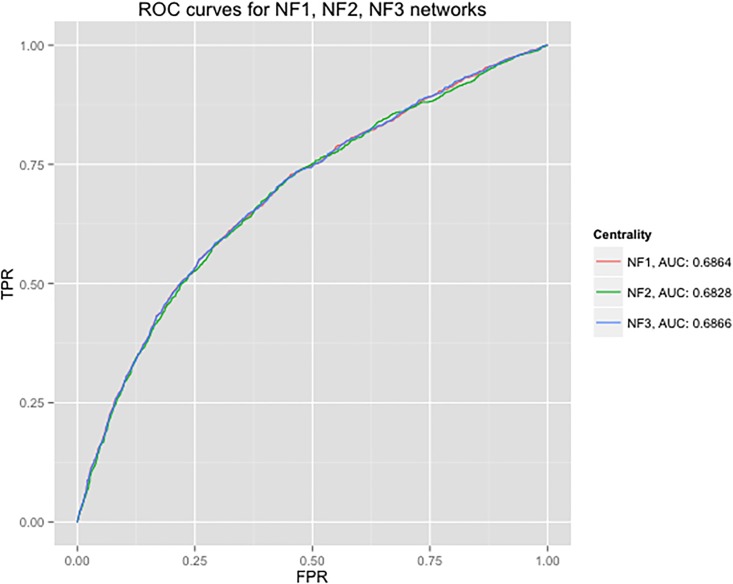
NF1,2,3_ROC: ROC curves for using various gene coexpression measures as biasing factors in DiffSLC. ROC curves of NF1, NF2, and NF3 networks are plotted along with their corresponding *β*, *ω*, and AUC.

To assess the differences in predictability of DiffSLC, a different gene expression dataset was utilized. Guan et.al. [30]’s experiment was chosen, which is performed on a different strain of yeast, but using the same Affymetrix GeneChip Array platform. The networks NF1, NF2, and NF3 were created using the Pearson correlation, the Spearman’s rank correlation, and the distance correlation of gene expression profiles, respectively, as the edge weights of the network N0. For the networks NF1, NF2, and NF3 corresponding to this dataset, Fig 5 shows the ROC comparison of calculating DiffSLC with the biased degree centrality (BDC) based on the ECC and the corresponding co-expression of the adjacent edges to a given node. The ROC was calculated using the same method as the previous analyses.

Fig 5 shows that similar to the case of the NT1,2,3 networks, the choice of coexpression measures has a negligible impact for the Guan et.al. data set. It is unclear as to which of the NF1,2,3 networks produced a better result than the others. Because the Guan et al. data set is a time series experiment with the global gene expression varying drastically when under influence of nonsense-mediated mRNA decay (NMD) [30], it is difficult to pick one coexpression measure as being a better choice solely based on the the gene expression profiles.

**Fig 6:**
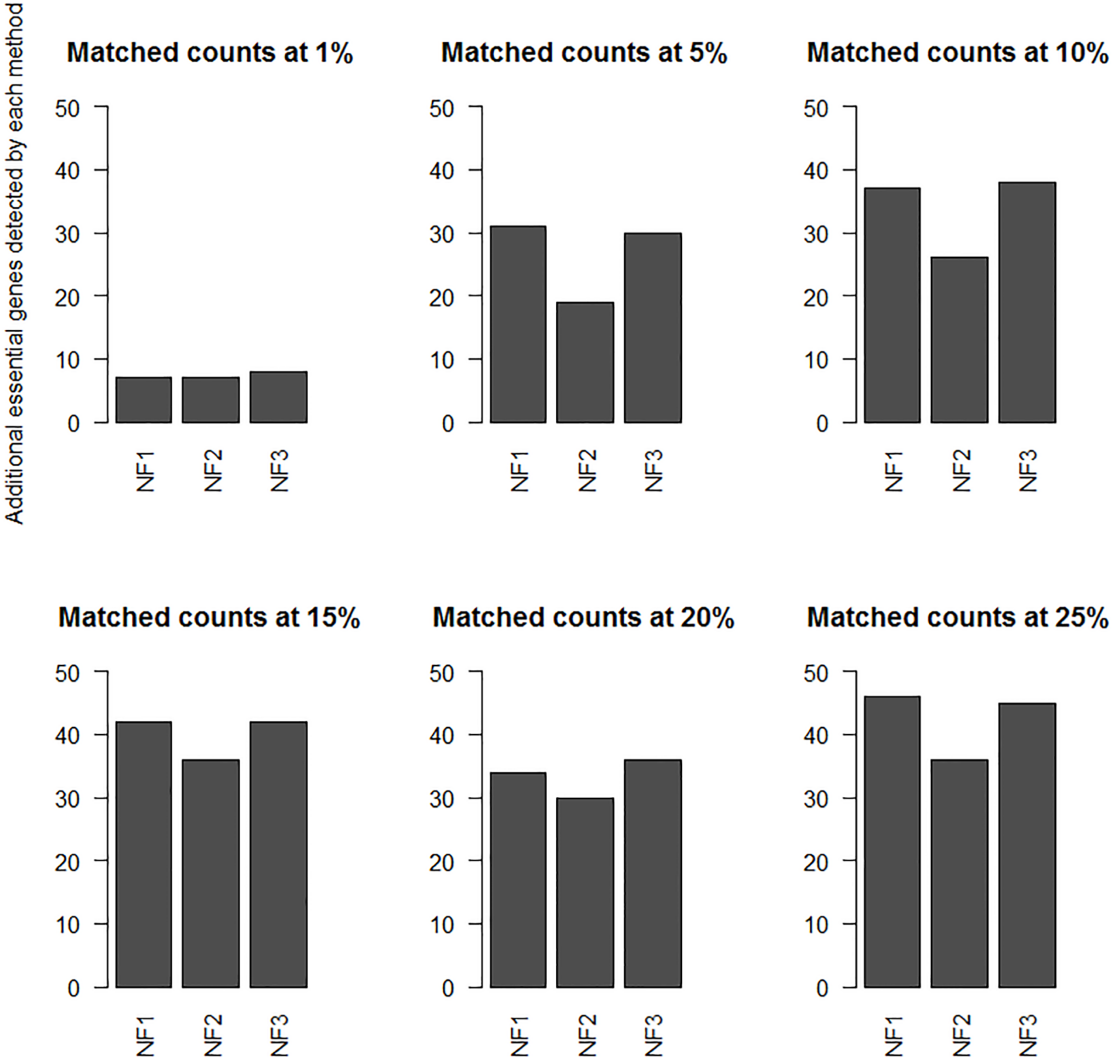
NF1,2,3_Top ranked proteins. Counts of unique essential genes ranked by various DiffSLC variants in the top 1%, 5%, 10%, 15%, 20%, and 25% are shown in the plot. The counts are only for a set of genes that were detected by one of the networks but not the others.

The top ranked nodes of the network ranked by DiffSLC were compared to estimate the success of the prioritization method. The performance of the rankings was evaluated by comparing the top ranked proteins against the known essential proteins, as shown in Fig 6. Results from Fig 5 suggest that the number of detected essential proteins may not be very different, therefore similar to the NT1,2,3 networks, only the unique matched counts at various percentage level are shown. These counts are the number of essential proteins detected by each ranking method in the top ranks that the other methods did not detect.

Fig 6 shows that at each of the percent cutoff levels, either the NF1 or the NF3 networks detect a marginally higher number of essential genes. To compare the NF1,2,3 network results against the NT1,2,3 network results with minimal variability, the NF3 network is chosen to evaluate the differences between two networks.

**Fig 7:**
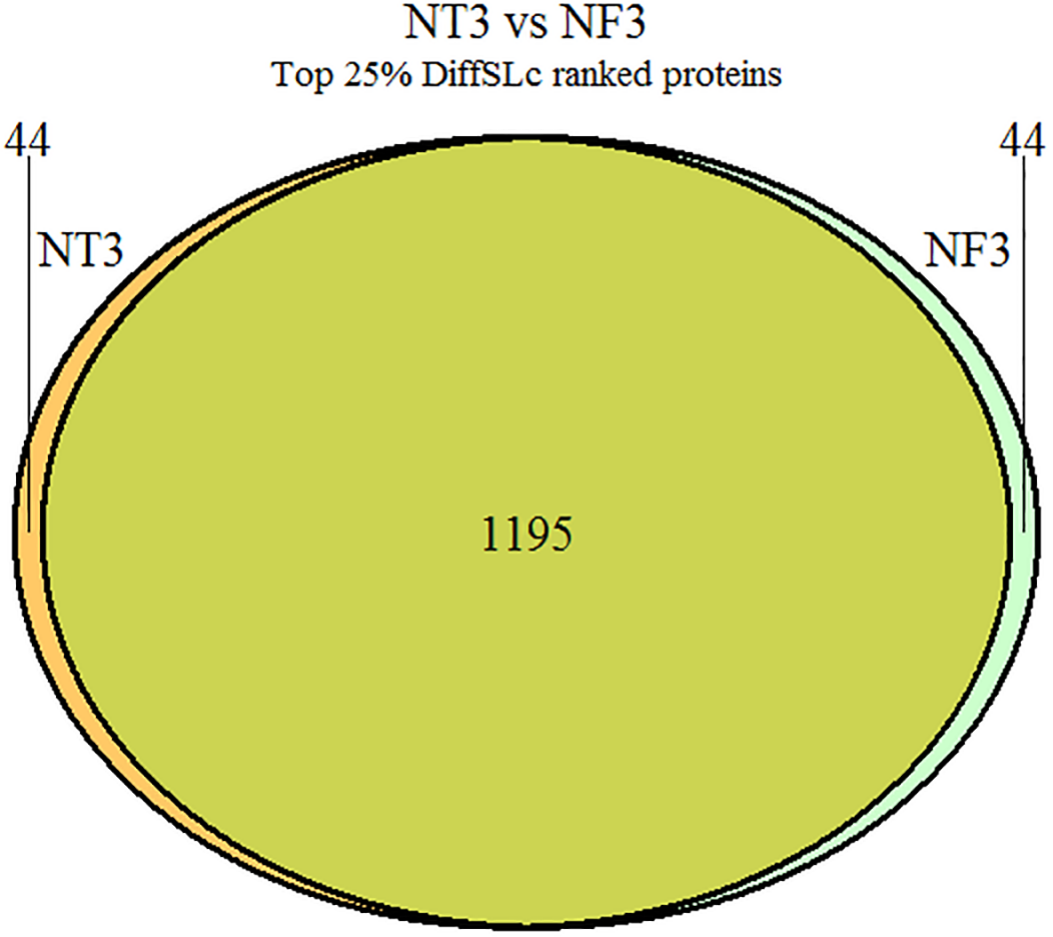
Number of shared top ranked proteins for NT3 and NF3. For top 25% of DiffSLC ranked proteins, different gene expression data resulted in different proteins being ranked higher. This Venn diagram shows the comparison of NT3 network to NF3 network’s ranked results. The full list of shared and different proteins is available in S1 Table.

The effect of choosing different gene expression dataset for centrality biasing was also notable. While there were roughly same number of essential proteins detected in top 25% ranked proteins, different expression datasets resulted in a number of different proteins being detected in those top ranked proteins. Fig 7 shows the number of shared and different proteins ranked in NT3 and NF3 networks.

### Evaluation of DiffSLC performance

**Fig 8:**
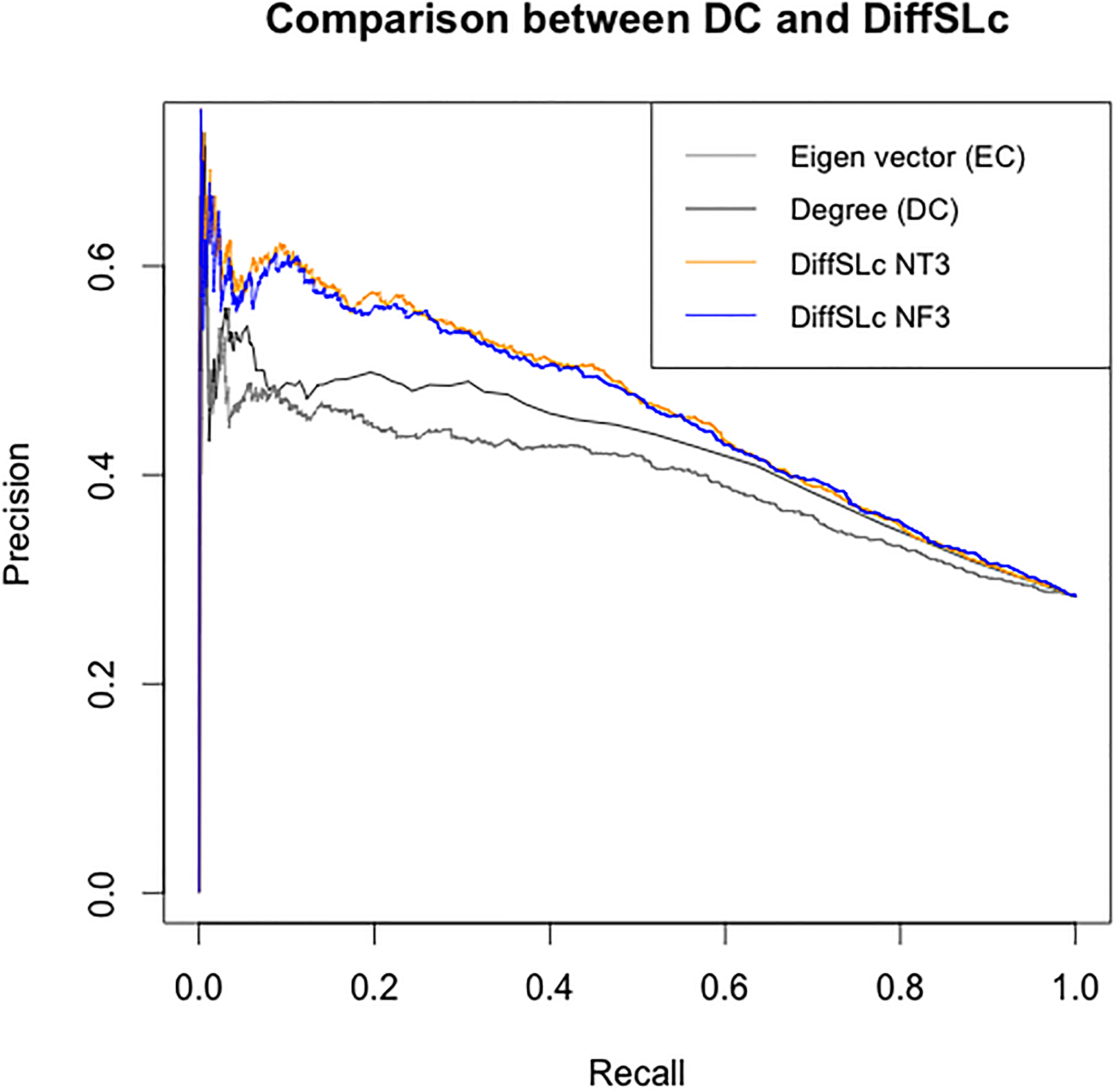
Precision-Recall curve comparing EC, DC, and DiffSLC. The precision-recall curves are plotted to show enhancement of the degree centrality by biasing coexpression, and combining the eigenvector of the adjacency matrix.

In addition to a better detection of essential proteins in the top-25% ranked proteins shown through the ROC curve evaluation, to assess the improvement resulting from combination of both the centrality and the biases, Fig 8 shows precision-recall curves of EC, DC, and DiffSLC. The higher the curve, the better the corresponding metric at discriminating between essential and non-essential proteins.

Fig 8 shows that results from the NT3 and the NF3 networks are similar to each other, and both are better than the DC and the EC measures alone.

## Discussion

DiffSLC is an effective computational method to discover essential proteins in a protein-protein interaction (PPI) network. It combines node and edge centrality methods with gene expression data to obtain improvements in detection of protein essentiality in yeast protein interaction networks.

### Comparison with other methods

There are at least three similar and often cited measures that also try to tackle the protein essentially prediction. These are PeC [39], Weighted Degree Centrality [40], and weighted degree applied to an active network construction [52]. We had reached out to the corresponding authors of these methods to obtain their source data and an implementation of their software; however, we had not received any response. The performance claims presented in the respective publications could not be verified as-is. Because the Weighted Degree Centrality was presented as a more generalized approach to PeC, and it had outperformed PeC based on their publication, the DiffSLC source code available at git.io/diffslc includes an implementation of Tang et.al.’s Weighted Degree Centrality. The comparison based on this implementation did not corroborate with the data published in the original publication, therefore the comparisons were ommited for Tang et.al.’s Weighted Degree Centrality based measures in this publication. The results of this comparison are still comparable by running the methods in DiffSLC source code.

There are two additional methods that showed promise in essentiality predictions for yeast: LBCC [19] method, and Plaimas et.al.’s support vector machine based [20] method. LBCC uses protein interaction data and combines it with protein complex interaction propensities to weigh a subset of interaction more or less than the other. This method shows noticeable improvement over methods utilizing only the protein interaction data sets. LBCC was tested on yeast and human interaction data. Although this method shows a concrete advancement in predicting yeast gene essentiality, the improvement of LBCC over traditional centrality methods is specifically due to known protein complexes and their propensities. Whether this method is advantageous in absence of high-quality protein interaction propensity data or not is unclear. A possible direction or comparison could be to perform *de novo* protein interaction prediction to estimate interaction propensities, and then check if those improve the performance of the LBCC measure. While useful, this exercise is beyond the scope of DiffSLC publication. In Plaimas et.al. [20], the authors have proposed a support vector machine based method which utilizes several biologically relevant features in addition to protein interaction to predict yeast gene essentiality. These features include number of codons, phyletic retention, base composition at silent site, and over twenty other features related to metaboloic networks. While this svm method with metabolic network features excels in the eukaryotic organisms it is tested in, its applicability in organisms whose metabolic network are not well-studied, is unknown. Whether a computationally predicated network would provide the same benefits claimed in the Plaimas publication is also unclear. That exercise might be a useful alternate direction to investigate in future publications.

While the methods that use various types of biological data such as cellular localizations of proteins, protein interaction affinities, and disease causing genes can often show improved essentiality prediction in eukaryotes and yeast, their results are dependent on having all the various types of biological experimental data for a specific organism that the essentiality prediction methods need to be applied to. Protein interaction and gene expression experiments are typically the only available data sets for many of those organisms, which makes a method that relies on expression and interaction experiments is widely applicable and worth a consideration. DiffSLC is designed to meet that criteria. If additional curated and verified biological data such as metabologic networks, gene regulatory networks, phyletic retention, gene silencing, etc. exist for an organism of interest, a relevant metric to incorporate those data sources can be investigated. An expansive review of recent advances in essentiality prediction methods for such circumstances was published in [17], which would be of a reader’s interest.

### Evaluating the results and performance of DiffSLC

Testing the accuracy of a network centrality based predictive ranking method requires a verified reference list to compare against the method results. Yeast is the only eukaryotic organism for which a genome-wide single gene knockout experiments and corresponding fitness data is publicly available. These results from *Saccharomyces* Gene Deletion Project (SGDP) [24, 36] have been curated as an essential genes list by Database of Essential Genes (DEG) [23, 37]. The Database of Interacting Proteins (DIP) [25, 26] as a source of experimentally determined interactions between proteins was used to create a protein interaction network. DIP provided a mapping between interacting proteins and their UniProtKB/SwissProt identifiers and Ensembl gene identifiers. These were matched against the annotations provided in Affymetrix Yeast Genome S98 array, which was used by the gene expression experiments utilized to implement expression biasing in DiffSLC. In order to create a network with verifiable relevant information, unmatched proteins were removed. The list of matched and unmatched DIP interactor proteins, along with relevant codes are made available at http://git.io/diffslc.

DiffSLC is able to better discriminate the essentiality due to three important features.

- DiffSLC keeps nodes with high degree centrality rank as-is, while incorporating low-degree ranking nodes that are essential by giving additional weight to high eigenvector centrality ranked nodes.
- DiffSLC promotes proteins involved in interactions where more interaction partners tend to interact with each other. This idea is captured through the edge clustering coefficient.
- DiffSLC ranks the interacting proteins that are result of highly coexpressed genes higher than the ones that are not. This usage assumes that essential genes would be highly coexpressed with several other genes, and that that effect would be noticed post translationally as well. For DiffSLC, gene coexpression provides additional ranking contribution from gene expression data.

For experiments with many observations of gene expression (e.g. several time points or experimental conditions), the distance correlation provides better estimate of gene coexpression. When many experimental conditions or time points in a long time course are observed, a safer assumption is to assume that the gene expression would increase or decrease at different conditions or time points for different genes, and that many genes may have cyclic spikes or dips in gene expression levels. A non-monotonic correlation is better suited to estimate the gene coexpression in such cases than a monotonic correlation measure. On the other hand, complex expression profiles are not detectable when coexpression is estimated based on just a few observations per gene. A monotonic correlation provides a reliable estimate for such experiments. This may explain the improved results of distance correlation in DiffSLC compared to either the Spearman’s rank correlation or the Pearson correlation.

DiffSLC estimate has two weighting parameters: *β* and *ω*. The *β* parameter scales contribution of gene coexpression values (*dCor*), which depend on the gene expression data; and the edge clustering coefficient (ECC), which depends on the graph topology derived from protein-protein interaction data. The success of a low *β* values suggest that for the context of biasing the degree centrality, the topological position of edges in reference to their neighbors is a stronger indicator of essentiality than the pair-wise correlation of the gene expression profiles. However, both are poor predictors of the essentiality on their own. On the other hand, the *ω* parameter weigh the eigenvector centrality (EC) of a node against the biased degree centrality computed using *dCor* and ECC. The success of a high *ω* value in the results indicate that in the context of supplementing BDC, the EC heavily contributes towards the improved essentiality detection of DiffSLC. The results also indicate the decisions that drive the choice of *β* and *ω* parameter values. For experiments with high number of replicates per experimental condition, the gene coexpression measure and edge clustering coefficient will have weights that are closer to each other, resulting in a *β* ≈ 0.5. The *ω* ≈ 0.9 produced the best results for all the variations of the networks that were considered. This may indicate that for a medium sized mixed networks created using gene co-expression and protein interaction evidence, the EC provides a high amount of gene essentiality relevance. Additional experiments would be necessary to prescribe a concrete range of values for each of the parameters given the various types and sizes of gene expression and protein interaction data.

When comparing the two networks *NT*3 and *NF*3 for the effect of different gene expression datasets, Fig 7 shows that approximately 6.8% ($\frac{88}{1295+44+44}$) of the detected essential genes were different based on the choice of gene expression data set. This may indicate that for a context-specific or experimental condition-specific detection of essential genes, a modified DiffSLC measure may be useful. Additional work would be necessary to validate this hypothesis. The precision-recall curve plotted higher in the plot represents a better binary classifier, because it suggests that the method is able to maintain low false positive numbers even with high false negative numbers (i.e. low recall—high precision cases). As the number of false negatives increase, the method represented by curve at the top of the plot is able to maintain higher precision than other methods. Looking at Fig 8, the top curves representing DiffSLC method suggest that essential protein detection is improved by combining the eigenvector centrality and a biased degree centrality. The EC and the DC individually perform worse than either of the DiffSLC curves plotted. This is because although the EC and DC detect several essential proteins in top ranked nodes of the network, they are non-overlapping low number of proteins. The DiffSLC metric is able to combine the results noticed in both. No clear evidence suggests a significant difference between the DiffSLC implementations in two different networks presented here.

The presented DiffSLC implementation is heavily dependent on the network topology, and hence the results are at most only as reliable as the network itself. Although this work utilizes a set of microarray experiments, the method is equally applicable to a next-generation sequencing data generated via an RNA-Seq platform. Ballouz et al. [53] have discussed the merit of utilizing RNA-Seq for generating reliable co-expression networks similar to microarray datasets. Iancu et al. [54] had shown that *de novo* coexpression networks constructed for tissues from two different mice strains using RNA-Seq experiments had a majority of the subnetworks that corresponded well to their microarray experiment counterparts. On the other hand, Giorgi et al. [55] and Han et al. [56] showed that the coexpression networks built by RNA-Seq and microarray experiments only had a small subset of similar subnetworks. The reliability and robustness of co-expression network construction is a topic better addressed elsewhere; however, the work reviewed above concluded that in an RNA-Seq experiment with a high number of samples (more than 20 according to [56]) and a high read depth (more than 10 million according to [56]), the coexpression networks would be generally reliable and relevant network analysis methods would produce useful results.

More than a general-purpose centrality method, DiffSLC proposes the use of experimentally relevant biases, and constraints geared towards specific networks. The same general framework of DiffSLC implementation can be utilized for biasing other graph centrality measures. For example, a network where some known critical nodes are most associated with the shortest-path betweenness centrality, the edge weights and path lengths can be weighted by edge-clustering coefficient (ECC), and also forced to meet weight or path length constraints. Biasing the betweenness centrality with such modifications would relax the shortest path computation to allow for longer paths that satisfy specific constraints, or eliminate shortest paths with low ECC (or gene coexpression level, if utilized). This would be a useful feature for use in the biological networks where the known biological information is not encoded in the network structure. Constraint driven analysis has already shown promise [57, 58], and a separate investigation will be required to understand the role of constraint-based centrality methods in protein essentiality prediction.

## Conclusion

DiffSLC is an effective computational method to discover essential proteins in a protein-protein interaction (PPI) network. It combines node and edge centrality methods with gene expression data to obtain improvements in detection of protein essentiality in yeast protein interaction networks. The effectiveness of DiffSLC was demonstrated using three variations of networks: (1) inclusion or exclusion of gene co-expression data, (2) impact of different coexpression measures, and (3) impact of different gene expression data sets. For a total of seven networks, DiffSLC was compared to other centrality measures using *Saccharomyces cerevisiae* protein interaction networks and gene expression data. When DiffSLC ranked genes were compared against the known essential genes from the *Saccharomyces* Gene Deletion Project, DiffSLC detected more essential proteins with a higher area under the ROC curve than other compared methods. This made DiffSLC a stronger alternative to other centrality methods for detecting essential genes using a protein-protein interaction network that obeys centrality-lethality principle.

## Supporting information

S1 Table Difference in top ranked proteins between NT3 and NF3 networks. DiffSLC ranked proteins based on using two different expression data sets. The results from each ranking are compared to find the differences in DiffSLC detected essential proteins.
(XLSX)

S2 Table AUC of the ROC based on choices of *ω* and *β*. This table shows impact of various *ω* and *β* value choices on the AUC of the ROC for DiffSLC applied to NT1, NT2, NT3, and NF1, NF2, NF3 networks.
(XLSX)
